# Supplementary material for: Nitro-Group-Containing Thiopeptide Derivatives as Promising Agents to Target Clostridioides difficile
Source: Pharmaceuticals (Basel). 2022 May 19;15(5):623. doi: 10.3390/ph15050623 (PMC9142968; doi:10.3390/ph15050623)
Supplement: Supplementary file 1 [file pharmaceuticals-15-00623-s001.zip › pharmaceuticals-1731086-supplementary.pdf]

Nitro-Group Containing Thiopeptide Derivatives as a Promising Agents to Target *Clostridioides difficile*

Dahyun Kim [1,†], Young-Rok Kim [2,†], Hee-Jong Hwang [1], Marco A. Ciufolini [3], Jusuk Lee [1], Hakyeong Lee [1], Shyaka Clovis [1], Sung Ji Jung [2], Young-Jin Son [1,*], Jin-Hwan Kwak [2,*]

[1]A&J Science Co., Ltd., 80 Chumbok Ro, Dong Gu, Daegu, 41061, Republic of Korea

[2] School of Life Science, Handong Global University, 558 Handong Ro, Heunghae-eup, Buk-gu, Pohang, 37554, Republic of Korea

[3] Department of Chemistry, University of British Columbia, 2036 Main Mall, Vancouver, BC, V6K 1Z1, Canada

Supporting Information

^{1}H and ^{13}C NMR Spectra

Table of Contents

Figure S1:^{1}H NMR Spectrum of compound **2** P3
Figure S2:^{13}C NMR Spectrum of compound **2** P4
Figure S3:^{1}H NMR Spectrum of compound **3** P5
Figure S4:^{13}C NMR Spectrum of compound **3** P6
Figure S5:^{1}H NMR Spectrum of compound **4** P7
Figure S6:^{13}C NMR Spectrum of compound **4** P8
Figure S7:^{1}H NMR Spectrum of compound **AJ-024** P9
Figure S8:^{13}C NMR Spectrum of compound **AJ-024** P10
Figure S9:HPLC chromatogram of compound **AJ-024** P11

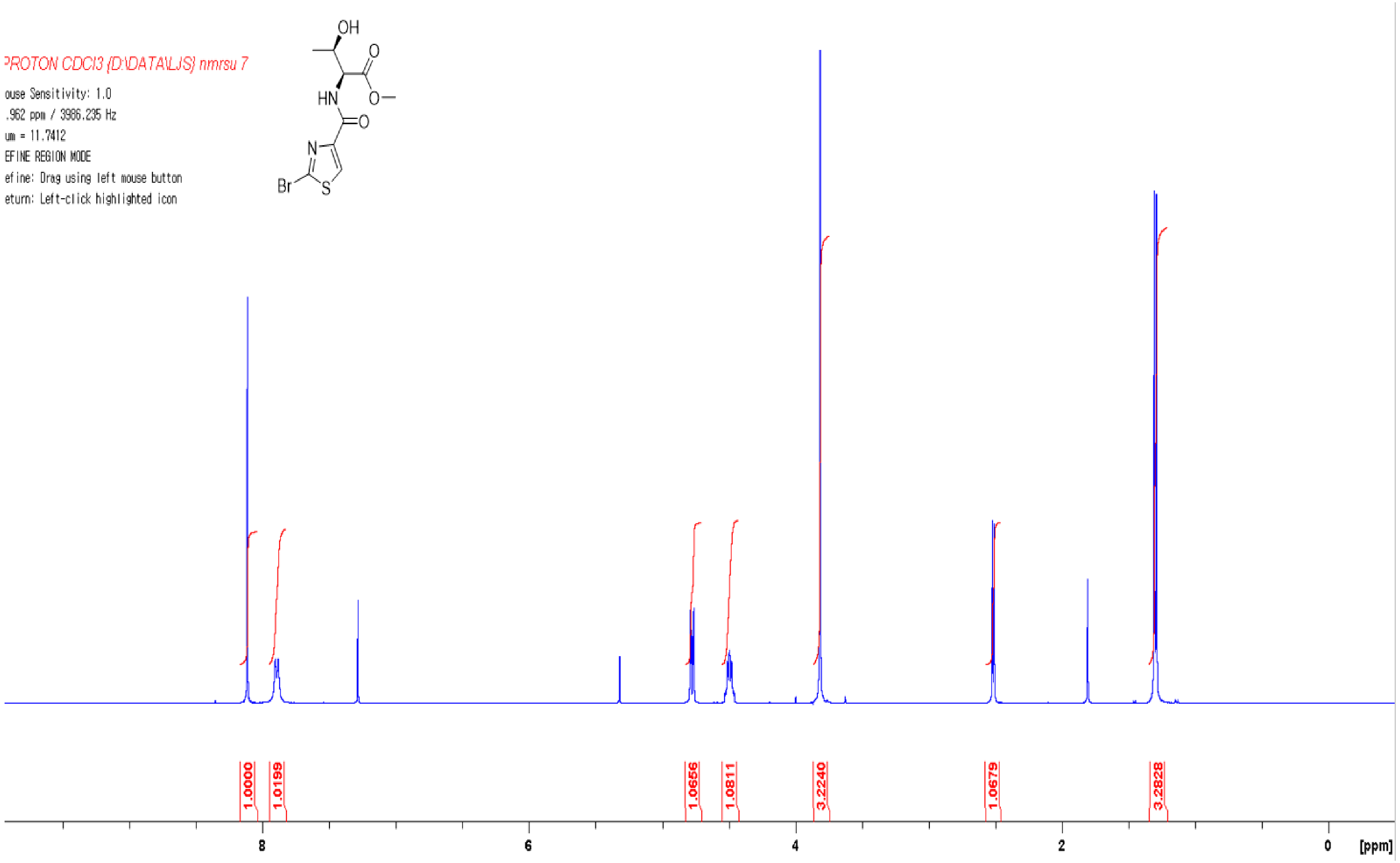

Figure S1:^{1}H-NMR Spectrum of compound **2** (400 MHz, $CDCl_3$)

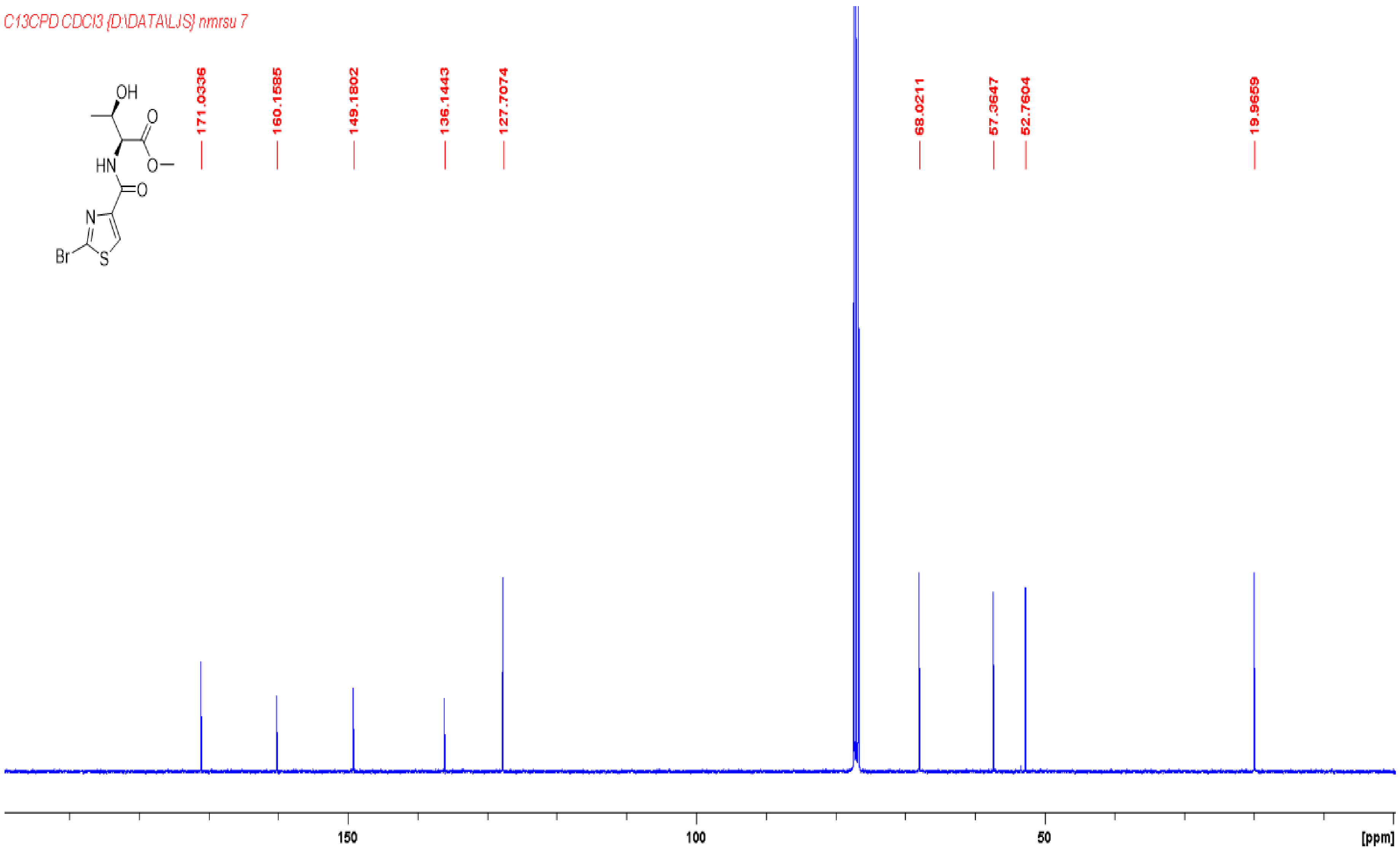

Figure S2:^{13}C-NMR Spectrum of compound **2** (101 MHz, $CDCl_3$)

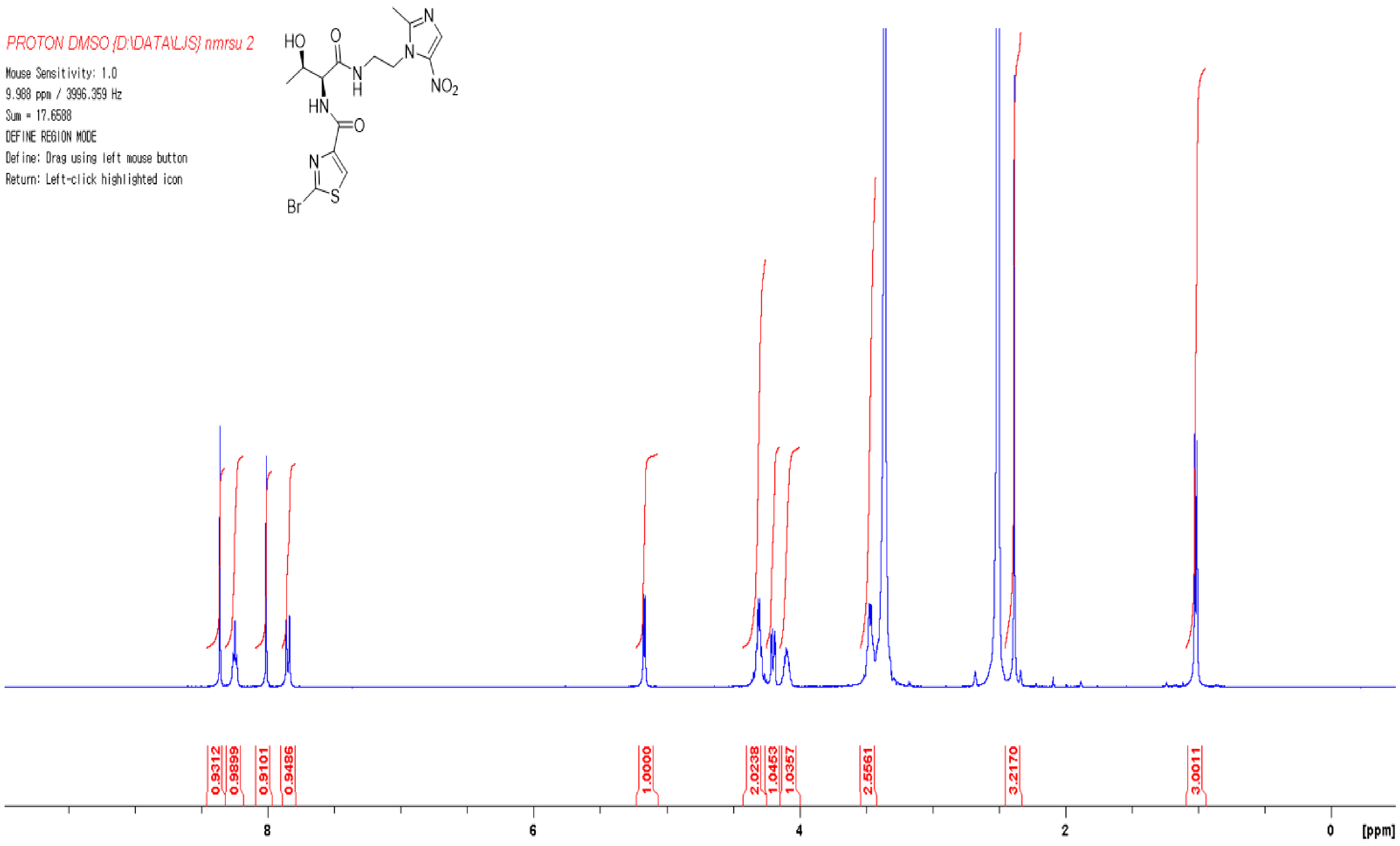

Figure S3:^{1}H-NMR Spectrum of compound **3** (400 MHz, DMSO-d_6)

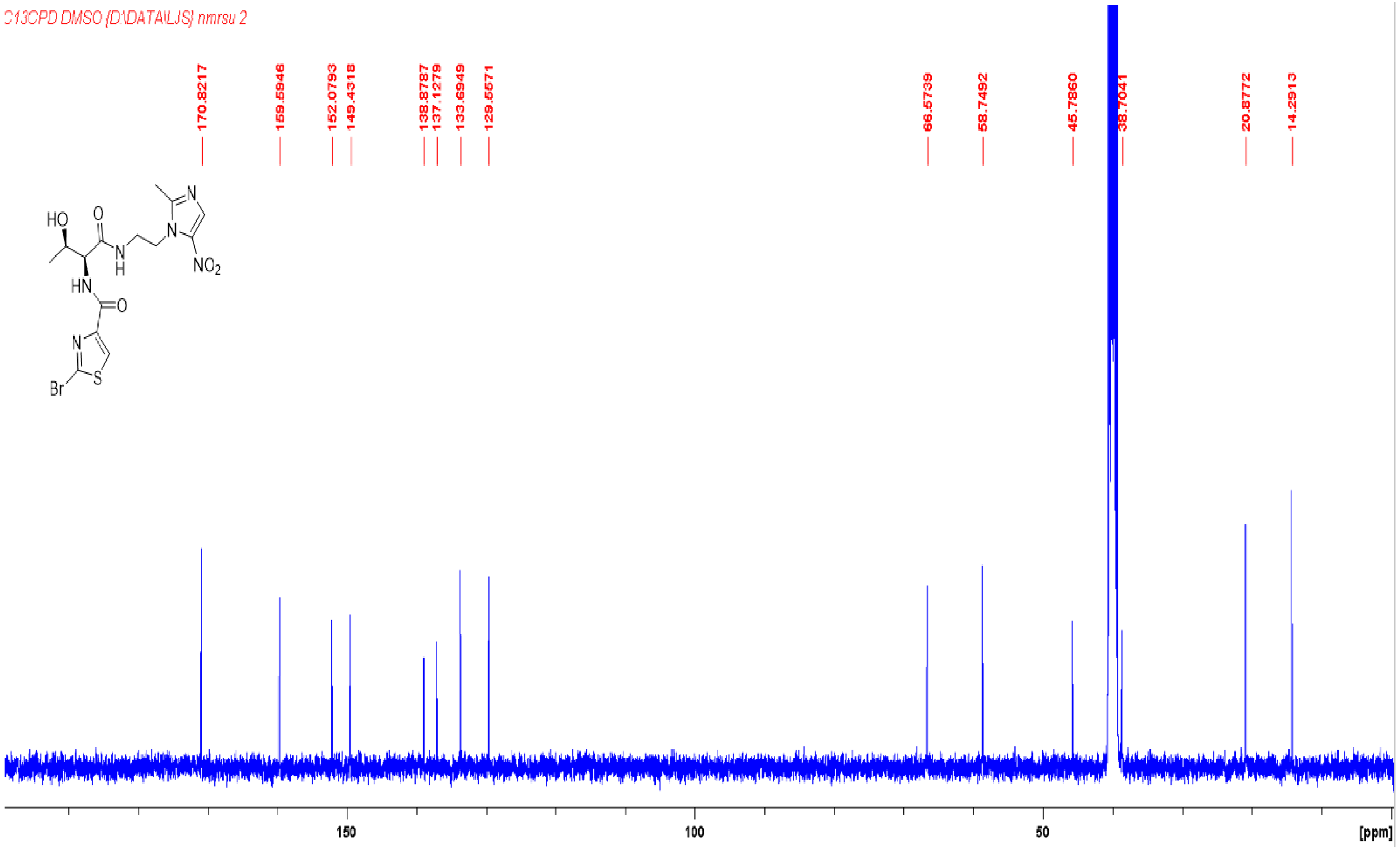

Figure S4: ^{13}C-NMR Spectrum of compound **3** (101 MHz, DMSO-*d*$_6$)

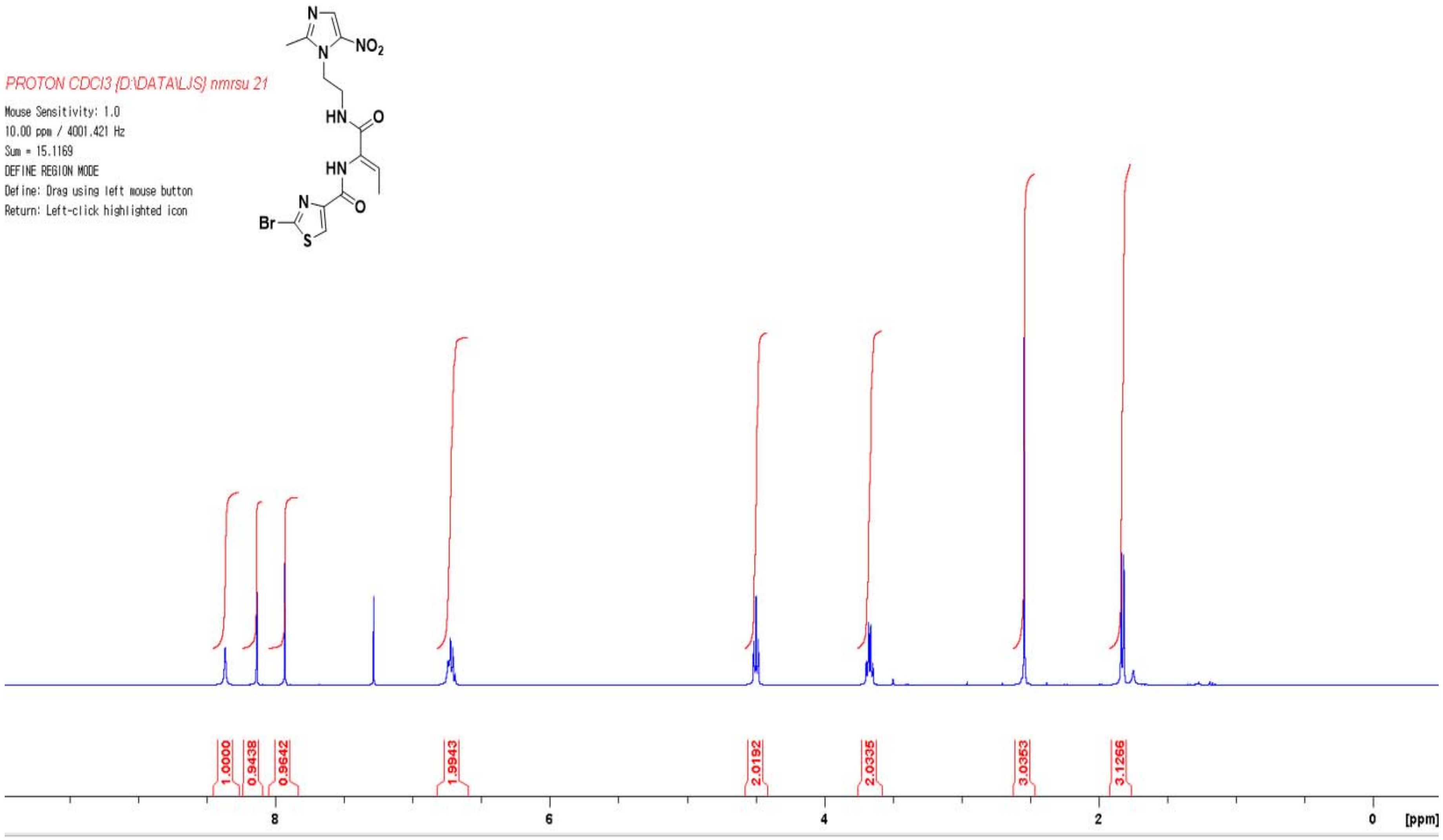

Figure S5:^{1}H-NMR Spectrum of compound **4** (400 MHz, $CDCl_3$)

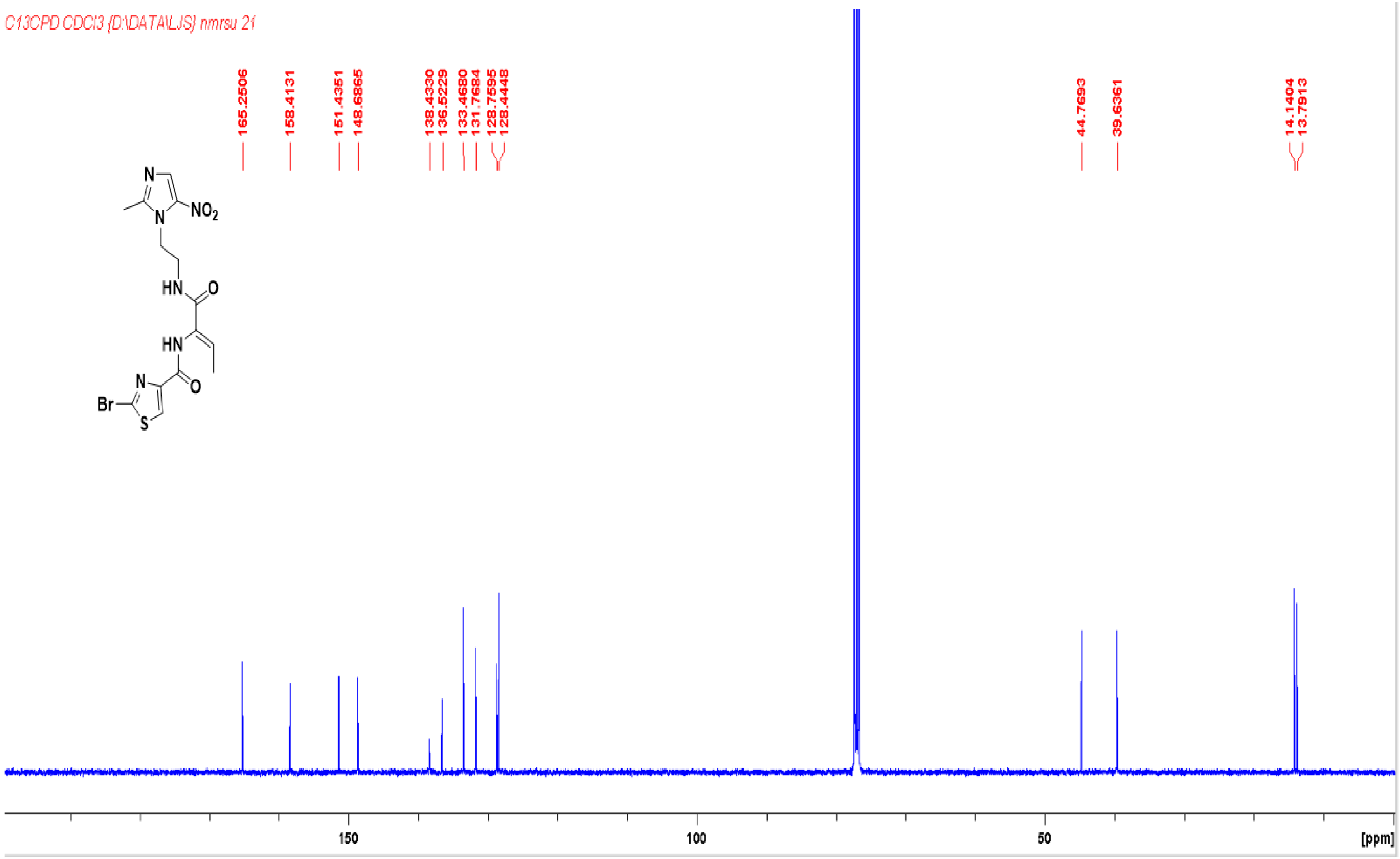

Figure S6: ^{13}C-NMR Spectrum of compound **4** (101 MHz, $CDCl_3$)

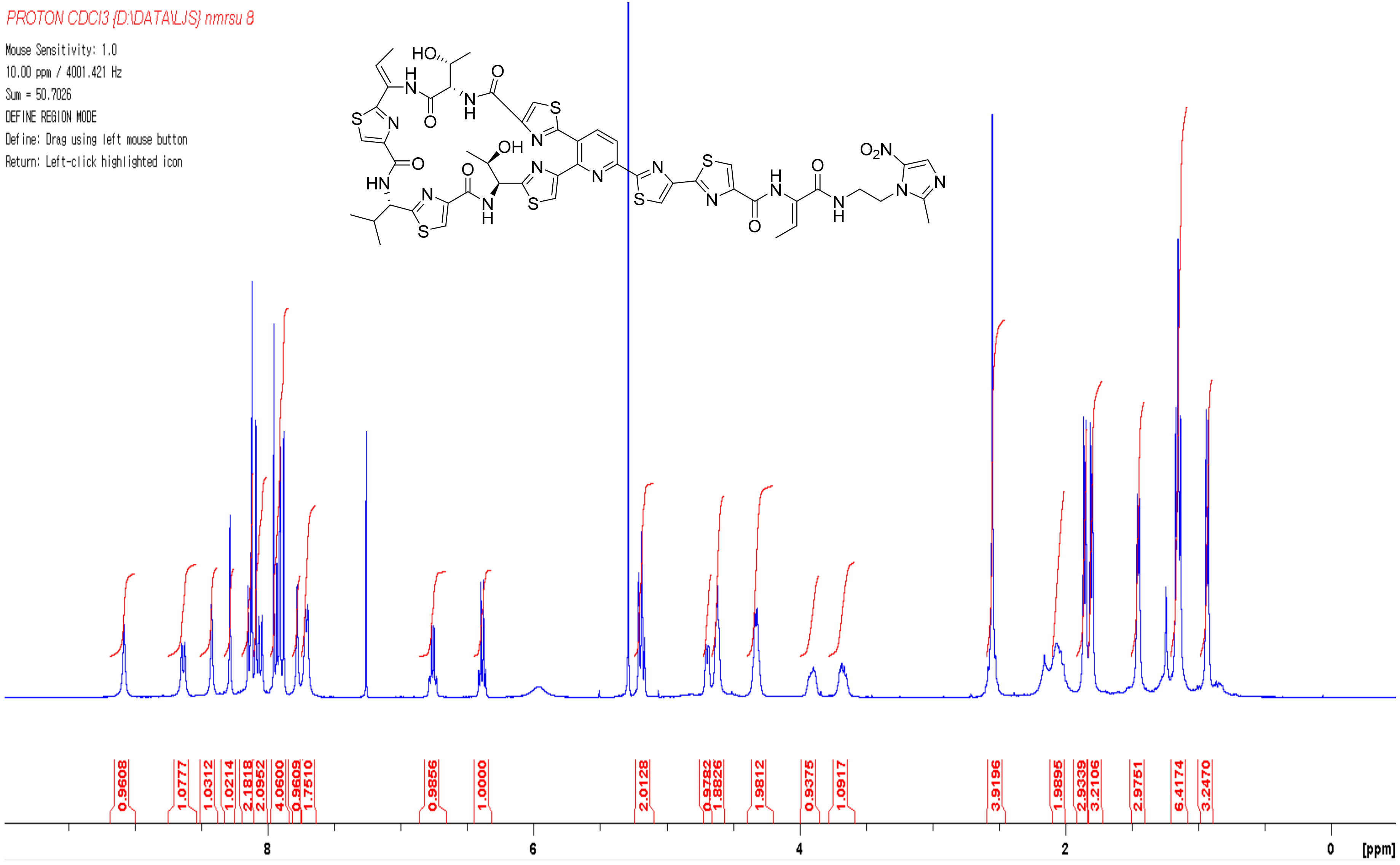

Figure S7: ^{1}H-NMR Spectrum of compound **AJ-024** (400 MHz, $CDCl_3$)

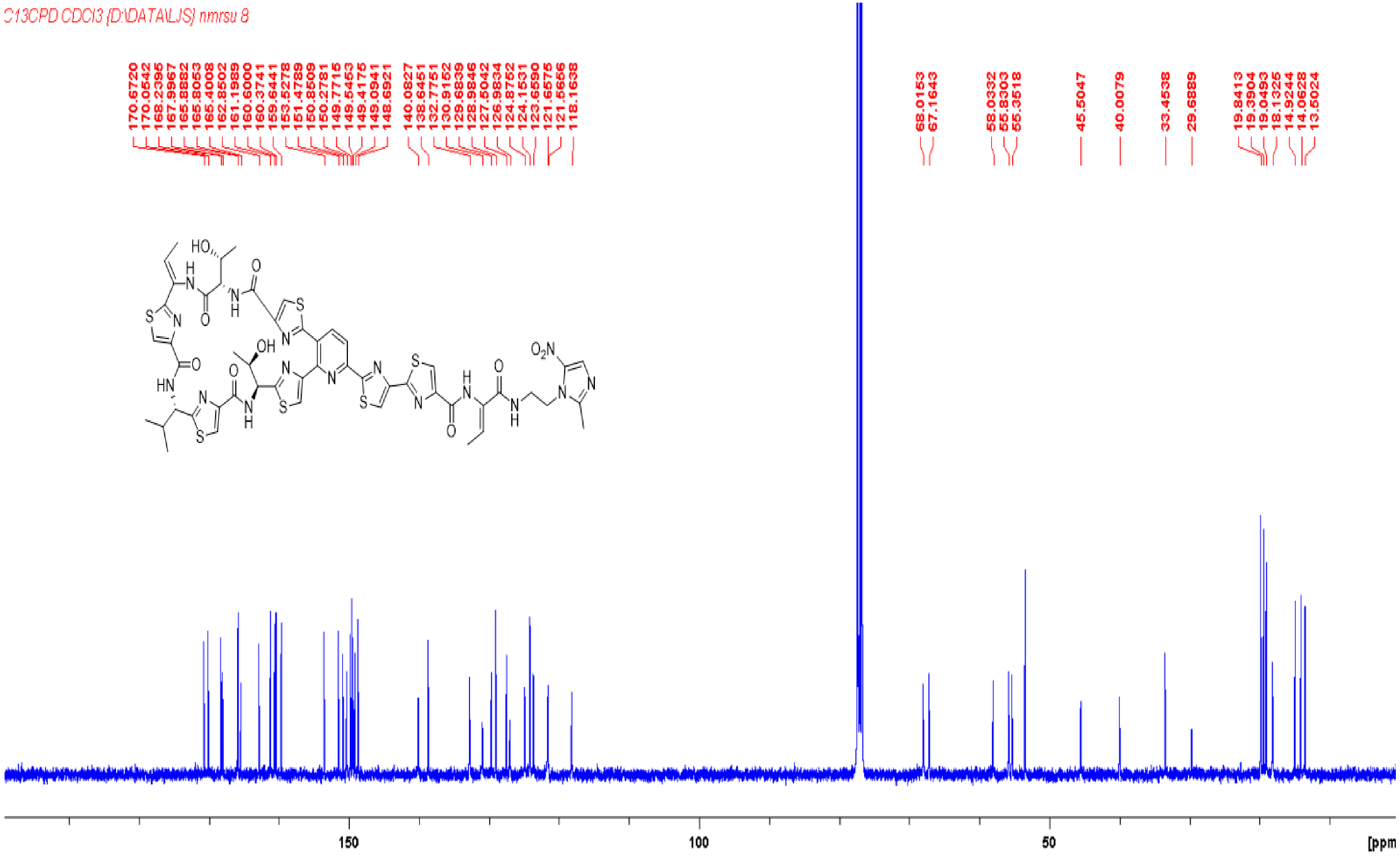

Figure S8: ^{13}C-NMR Spectrum of compound **AJ-024** (101 MHz, $CDCl_3$)

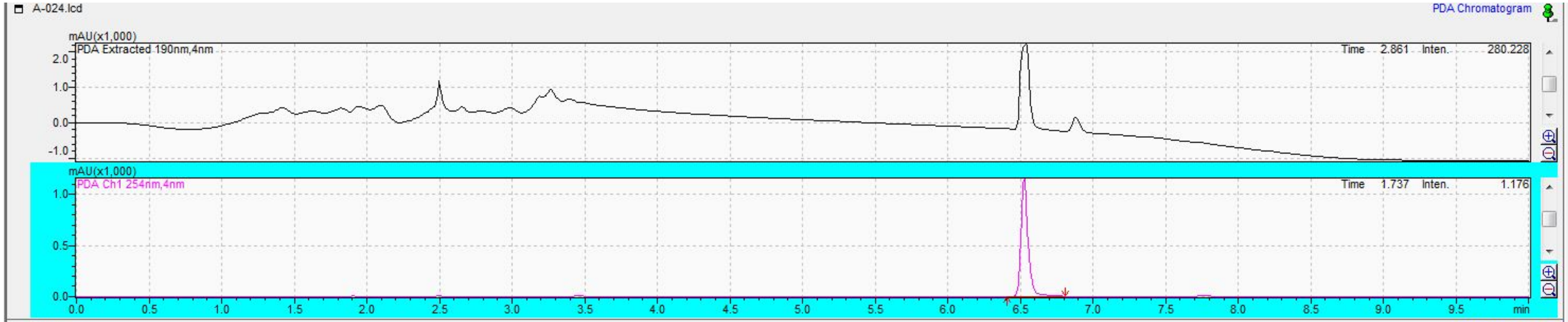

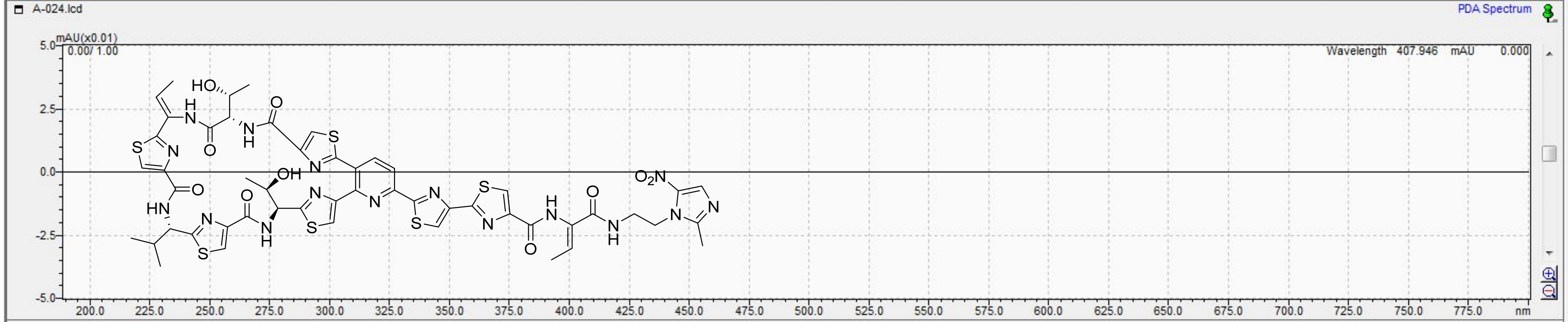

A-024.lcd

PDA Peak Table

Peak#	Ret. Time	Area	Height	Mark	Conc.	Unit	ID#	Name	Area%
1	6.524	4033450	1150800	M	0.000				100.000
Total		4033450	1150800		0.000				100.000

Extracted 190nm | Ch1 254nm

Figure S9:HPLC chromatogram of compound **AJ-024**
